# A phenome-wide association study of a lipoprotein-associated phospholipase A_2_ loss-of-function variant in 90 000 Chinese adults

**DOI:** 10.1093/ije/dyw087

**Published:** 2016-06-14

**Authors:** Iona Y Millwood, Derrick A Bennett, Robin G Walters, Robert Clarke, Dawn Waterworth, Toby Johnson, Yiping Chen, Ling Yang, Yu Guo, Zheng Bian, Alex Hacker, Astrid Yeo, Sarah Parish, Michael R Hill, Stephanie Chissoe, Richard Peto, Lon Cardon, Rory Collins, Liming Li, Zhengming Chen, on behalf of the China Kadoorie Biobank Collaborative Group

**Affiliations:** 1 Clinical Trial Service Unit & Epidemiological Studies Unit (CTSU), Nuffield Department of Population Health, University of Oxford, UK; 2 GlaxoSmithKline (GSK) Medicines Research Centre, GSK, Stevenage, UK, Research Triangle Park, NC, USA and King of Prussia, PA, USA; 3 Chinese Academy of Medical Sciences, Dong Cheng District, Beijing, China and; 4 Department of Epidemiology & Biostatistics, Peking University Health Science Centre, Beijing, China

**Keywords:** Lp-PLA_2_, genetic association, vascular disease, phenome-wide, China

## Abstract

**Background:** Lipoprotein-associated phospholipase A_2_ (Lp-PLA_2_) has been implicated in development of atherosclerosis; however, recent randomized trials of Lp-PLA_2_ inhibition reported no beneficial effects on vascular diseases. In East Asians, a loss-of-function variant in the *PLA2G7* gene can be used to assess the effects of genetically determined lower Lp-PLA_2_.

**Methods:** *PLA2G7* V279F (rs76863441) was genotyped in 91 428 individuals randomly selected from the China Kadoorie Biobank of 0.5 M participants recruited in 2004–08 from 10 regions of China, with 7 years’ follow-up. Linear regression was used to assess effects of V279F on baseline traits. Logistic regression was conducted for a range of vascular and non-vascular diseases, including 41 ICD-10 coded disease categories.

**Results:** *PLA2G7* V279F frequency was 5% overall (range 3–7% by region), and 9691 (11%) participants had at least one loss-of-function variant. V279F was not associated with baseline blood pressure, adiposity, blood glucose or lung function. V279F was not associated with major vascular events [7141 events; odds ratio (OR) = 0.98 per F variant, 95% confidence interval (CI) 0.90-1.06] or other vascular outcomes, including major coronary events (922 events; 0.96, 0.79-1.18) and stroke (5967 events; 1.00, 0.92-1.09). Individuals with V279F had lower risks of diabetes (7031 events; 0.91, 0.84-0.98) and asthma (182 events; 0.53, 0.28-0.98), but there was no association after adjustment for multiple testing.

**Conclusions:** Lifelong lower Lp-PLA_2_ activity was not associated with major risks of vascular or non-vascular diseases in Chinese adults. Using functional genetic variants in large-scale prospective studies with linkage to a range of health outcomes is a valuable approach to inform drug development and repositioning.

Key Messages

- Genetically-determined lower Lp-PLA_2_ activity was not associated with lower risks of major vascular diseases in a large Chinese population, consistent with findings from clinical trials of Lp-PLA_2_-lowering therapy.
- There was also no association between the *PLA2G7* V279F loss-of-function variant and a phenome-wide range of non-vascular diseases, and several traits including blood pressure, adiposity and lung function.
- Functional genetic variants in large-scale prospective studies with linkage to a wide range of health outcomes can be used to predict the potential beneficial and harmful effects of novel therapeutic strategies before undertaking costly clinical trials.

## Introduction

Lipoprotein-associated phospholipase A_2_ (Lp-PLA_2_), also known as platelet-activating factor acetylhydrolase (PAF-AH), is an enzyme expressed by activated inflammatory cells in atherosclerotic lesions, and found at high levels in unstable and ruptured plaques.^1^ Lp-PLA_2_ circulates in plasma bound predominantly to low-density lipoprotein (LDL) particles.^2^ Although Lp-PLA_2_ produces the pro-inflammatory mediators lysophosphatidylcholine and oxidized free fatty acids through hydrolysis of oxidized phospholipids on LDL, it also has anti-inflammatory activity through degradation of platelet-activating factor,^3^ and its biological role in the initiation and progression of atherosclerosis is uncertain.^4^ The Lp-PLA_2_ inhibitor darapladib reduces Lp-PLA_2_ activity by >60%;^5^ however, two phase III trials in 28 854 patients with stable coronary heart disease (CHD) or acute coronary syndrome (ACS), with about 3 years of treatment, failed to establish a protective role of darapladib for prevention of further major vascular disease.^6^^,^^7^

Several epidemiological studies in mainly Western populations have examined the associations of Lp-PLA_2_ mass and activity with risk of vascular diseases. A meta-analysis of 79 036 individuals from 32 prospective studies reported that one standard deviation higher Lp-PLA_2_ activity was associated with 8–16% higher risk of occlusive vascular disease, after adjusting for conventional risk factors, with the effect on CHD being similar in magnitude to that of LDL-cholesterol or systolic blood pressure (SBP).^8^ However, a study of 19 037 individuals with established occlusive vascular disease found no association between Lp-PLA_2_ activity and coronary events after more extensive adjustment for lipids,^9^ casting doubt on a causal role of Lp-PLA_2_ in CHD.

Functional genetic variants can be used to assess the causal role of proteins such as Lp-PLA_2_, and their potential value as therapeutic targets, in a manner analogous to a randomized controlled trial.^10^ A c.835G > T (amino acid substitution V279F) variant in the *PLA2G7* gene encoding Lp-PLA_2_ inactivates the enzyme, resulting in about 50% lower Lp-PLA_2_ activity for each copy of the loss-of-function variant.^11–13^*PLA2G7* V279F is rare in Europeans^14^ but relatively common in East Asian populations, with the frequency ranging from ∼5% in Chinese^13^ to 17% in Japanese.^15^ However, previous studies of *PLA2G7* V279F with vascular diseases in East Asians have produced conflicting findings. Two meta-analyses, each with about 3600 cases with some overlap, reported no apparent association with CHD risk.^16^^,^^17^ Subsequently, a study in Koreans reported a 20% lower risk of CHD associated with V279F among men (3700 cases) but no effect in women (1130 cases).^18^

Although Lp-PLA_2_ activity might play a role in multiple biological pathways, studies examining the role of *PLA2G7* V279F in diseases other than CHD, including other vascular diseases, are limited. We present findings from a large-scale study of over 90 000 adults from the China Kadoorie Biobank (CKB) prospective cohort, with health record linkage to a range of health outcomes. We previously reported a summary of the association of *PLA2G7* V279F with vascular diseases in the CKB.^19^ This study reports in detail the effects of the *PLA2G7* V279F loss-of-function variant on pre-defined major vascular diseases as hypothesis-testing of the randomized trials of inhibition of Lp-PLA_2_, and further examines in a hypothesis-free approach the associations of *PLA2G7* V279F with a phenome-wide range of other disease outcomes and traits.

## Methods

### Study population

The CKB design and methods are reported in detail elsewhere.^20^^,^^21^ Overall, 512 891 adults aged 30–79 years were enrolled during 2004–08 from 10 rural and urban regions in China. The baseline survey included a questionnaire on socio-demographic and lifestyle factors and medical history. Physical measurements included anthropometry, blood pressure and lung function. A 10-ml EDTA non-fasting blood sample was collected for on-site testing for blood glucose level (SureStep Plus meter) and long-term storage. Study procedures and staff training were standardized across regions. Local, national and international ethics approvals were obtained and all participants provided written informed consent for long-term follow-up through their health records.

### PLA2G7 *V279F genotyping*

DNA was extracted from 800 μl stored buffy coat using a magnetic bead purification method (KingFisher^TM^ Flex Magnetic Particle Processors). A 384-SNP array (Illumina® GoldenGate Genotyping Assay) including rs76863441 (*PLA2G7* V279F) was used to genotype 95 680 randomly selected samples. The genotyping success rate for rs76863441 was 99.99%. Following quality control, 2483 samples were excluded based on call rate < 98% (*n* = 2215), sex mismatch (*n* = 118), potential sample linkage errors (*n* = 149) and excess heterozygosity (*n* = 1). Pair-wise identity by descent was used to identify first-degree relatives (kinship ≥ 0.1875) within study regions. Within the dataset, 22% had at least one first-degree relative, and 1683 individuals were excluded so that family groups were all equally intra-related (i.e. all first-degree relatedness was restricted to groups of multiple siblings or of one parent plus one or more children). Furthermore, individuals outside the age range 30–79 years (*n* = 66) or with missing genotype data (*n* = 20) were excluded. After these exclusions, 91 428 individuals were used for all primary analyses in the current study (eFigure 1, available as Supplementary data at *IJE* online.). A subset of 82 459 individuals, used for sensitivity analyses and estimates of allele frequency and Hardy-Weinberg equilibrium, resulted from further excluding 8969 individuals, to leave no remaining first-degree relatedness.

### Long-term follow-up

Vital status and incidence of disease events were recorded using electronic linkage of each participant’s unique national identification number with established registries for morbidity (stroke, CHD, cancer and diabetes) and mortality in each locality, and a nationwide health insurance system. Registry data collected included scanned copies of official death certificates and original hospital disease reporting cards. Health insurance reports included detailed information (e.g. disease description, International Statistical Classification of Diseases and Related Health Problems 10th Revision [ICD-10] code and procedure/examination codes) about each hospital admission (one region also provided some outpatient data). Events related to major chronic diseases [stroke, CHD, diabetes, chronic obstructive pulmonary disease (COPD) and cancer] were carefully reviewed and standardized. By 1 January 2014, after a median of 7.2 years’ follow-up, 223 634 ICD-10 coded events, including 4585 deaths, were recorded among the 91 428 individuals in the present study, and 411 (0.4%) were lost to follow-up.

### Main outcome measures

The pre-specified primary outcome was incident major vascular events (MVE: vascular death, myocardial infarction, stroke). Secondary vascular outcomes were incident major coronary events (MCE: CHD death, myocardial infarction), major occlusive events (CHD death, myocardial infarction, ischaemic stroke), myocardial infarction, total stroke, ischaemic stroke, haemorrhagic stroke and vascular death. For all vascular outcomes, controls excluded individuals reporting a history of CHD, stroke or transient ischaemic attack at baseline, or incident MVE. Tertiary outcomes were diabetes and COPD, including both prevalent (previous history or screen-detected^22^^,^^23^) and incident cases and incident chronic kidney disease, liver disease, inflammatory disease, cancer and non-vascular death. Controls excluded individuals reporting a history of that disease at baseline. Incident events in the range ICD-10: A00-N99 were grouped into 41 distinct categories, largely following the ICD-10 classification (events outside this range included external causes and were not considered relevant to the present study). For these ICD-10 categorized outcomes, no exclusions for prevalent diseases were made from controls. For all outcomes, no exclusions for prevalent diseases were made from cases, i.e. not all cases were new onset. There was overlap between the clinically defined primary, secondary and tertiary outcomes, and the ICD-10 categorized outcomes. All disease outcomes were ascertained through death and disease registries and health insurance records. Disease outcomes were pre-specified in a detailed analysis plan and are described in the supplementary material (available as Supplementary data at *IJE* online). Selected continuous traits measured at baseline were also assessed.

### Statistical analyses

Baseline characteristics were standardized to the sex, age and region distribution of the study population, and compared across genotypes by a χ^2^-test for categorical measures or by analysis of variance for continuous measures. The association of *PLA2G7* V279F with continuous outcomes was assessed by linear regression, and disease outcomes by logistic regression, with an additive [per minor (F) allele] genetic model adjusting for sex, region, age as a continuous variable and relatedness using a robust sandwich estimator method,^24^ which may occasionally result in non-convergence of the model. Based on approximately 7000 incident cases, the study had over 90% power to detect a 20% lower risk of MVE per minor (F) allele (frequency = 0.05) at *P* < 0.01. Subgroup analyses were performed for the primary outcome, by sex, age group, region and ever regular smoking and current regular alcohol drinking status. Sensitivity analyses were performed for primary, secondary and tertiary outcomes, stratified by region without adjusting for relatedness, and using the subset of unrelated individuals. Exploratory analyses involved the addition of participants reported as undergoing revascularization procedures, or the addition of prevalent cases in combination with incident events, to the main vascular endpoints. *P*-values were presented unadjusted for multiple testing, but the threshold for significance at *P* < 0.05 was calculated using a standard Bonferroni correction by dividing 0.05 by the number of tests in each category of primary, secondary, tertiary and phenome-wide endpoints, or continuous traits. Analyses used SAS^®^ version 9.3 (SAS Institute Inc.).

## Results

### Participant characteristics and genotype distribution

Among the 91 428 study participants, the mean age at baseline was 51 years, 40% were men and 59% were from rural regions (Table 1). Previous history of physician-diagnosed CHD was reported by 3%, stroke or transient ischaemic attack by 2%, diabetes by 6% and hypertension by 12% of participants. Use of antihypertensive medication or statins was reported by 5% and 0.2% of participants, respectively. Baseline characteristics were generally similar between the whole CKB cohort of 512 891 participants and the randomly selected genotyped sample. Overall, 9691 (10.6%) participants had at least one copy of the loss-of-function variant. Participant baseline characteristics did not vary by *PLA2G7* V279F genotype, except for modest differences in the proportions from urban regions and reporting regular alcohol consumption, and mean physical activity level (Table 1).

**Table 1.** Characteristics of study participants by *PLA2G7* V279F genotype

Characteristic^a^	All CKB participants (*n* = 512 891)	Participants in the genetic sub-study (*n* = 91 428)	*PLA2G7* V279F genotype^b^			*P*-trend
			VV (*n* = 81 737)	VF (*n* = 9408)	FF (*n* = 283)	
Demographic						
Age (years)	51.5 (10.7)	51.4 (10.6)	51.4 (10.5)	51.3 (10.5)	52.6 (10.5)	0.12
Female (%)	59.0	59.7	59.7	60.1	61.8	0.56
Urban (%)	44.1	40.9	41.1	39.6	38.5	0.01
High school education or above (%)	21.0	19.6	19.5	19.9	19.1	0.76
Income > 20,000 yuan/year (%)	42.7	41.3	41.3	42.1	37.9	0.27
Previous disease						
History of hypertension (%)	11.6	11.5	11.5	11.6	11.3	0.92
History of coronary heart disease (%)	3.0	2.9	2.9	3.0	2.7	0.96
History of stroke or transient ischaemic attack (%)	1.7	1.7	1.7	1.5	2.5	0.23
History of diabetes (%)	5.9	5.9	6.0	5.4	5.8	0.08
Cardiovascular risk factors						
Physical activity (MET-h/day)	21.1 (13.9)	21.6 (14.0)	21.6 (12.0)	21.3 (12.0)	22.5 (12.0)	0.02
Ever regular smoker (%)	32.4	32.0	31.9	32.7	30.9	0.62
Regular drinker (%)	14.8	14.7	14.5	16.1	14.0	<0.0001
Medication use						
Antihypertensive therapy (%)	4.8	4.8	4.8	4.8	6.1	0.60
Statins (%)	0.2	0.2	0.2	0.2	0.4	0.79

^a^Values are mean (standard deviation) unless otherwise stated.

^b^All comparisons are adjusted for age, sex and region, except age (adjusted for sex and region), female status (adjusted for age and region) and urban status (adjusted for age and sex).

Assessed using the reduced dataset excluding first-degree relatedness, *PLA2G7* V279F frequency was 5% overall, but varied from 3% to 7% by region (*P*-heterogeneity < 0.0001), and genotype distribution within each of the 10 regions did not deviate from Hardy-Weinberg equilibrium (eTable 1, available as Supplementary data at *IJE* online).

### Association of PLA2G7 V279F with continuous traits

There were no differences by genotype in baseline physical measurements, including blood pressure, adiposity and lung function, after adjustment for sex, age, region, relatedness and multiple testing (Table 2). Random blood glucose level was assessed among participants not reporting a previous history of diabetes and was not associated with genotype.

**Table 2.** Association of *PLA2G7* V279F with continuous traits

		Mean (SE) by *PLA2G7* V279F genotype				
Outcome^a^	No. of participants	VV	VF	FF	Beta (SE) per minor (F) allele	*P*-trend^c^
Systolic blood pressure (mmHg)	91 428	131.3 (0.08)	131.9 (0.22)	130.3 (1.20)	0.43 (0.21)	0.04
Diastolic blood pressure (mmHg)	91 428	78.3 (0.04)	78.4 (0.13)	77.9 (0.70)	0.12 (0.12)	0.31
Body mass index (kg/m^2^)	91 428	23.7 (0.01)	23.7 (0.03)	23.5 (0.19)	−0.03 (0.03)	0.32
Waist-hip ratio (%)	91 428	88.1 (0.03)	88.0 (0.07)	88.4 (0.40)	−0.02 (0.07)	0.67
Random blood glucose (mmol/l)^b^	87 631	5.9 (0.01)	5.9 (0.02)	6.0 (0.12)	−0.02 (0.02)	0.28
FEV_1_ (litre)	91 428	225.6 (0.17)	226.1 (0.47)	222.6 (2.80)	0.32 (0.47)	0.49
FVC (litre)	91 428	265.1 (0.19)	265.4 (0.53)	261.9 (3.02)	0.05 (0.52)	0.92
FEV_1_/FVC ratio (%)	91 428	85.2 (0.03)	85.3 (0.08)	85.1 (0.49)	0.07 (0.08)	0.34

SE, standard error; FEV, forced expiratory volume; FVC, forced vital capacity.

^a^All analyses are adjusted for age, sex, region and relatedness.

^b^Assessed in participants not reporting a previous history of diabetes.

^c^*P*-trend not adjusted for multiple testing. Bonferroni correction based on eight tests would result in a threshold of 0.006 (*P* = 0.05/8).

### Association of PLA2G7 V279F with vascular diseases

**Figure 1.**
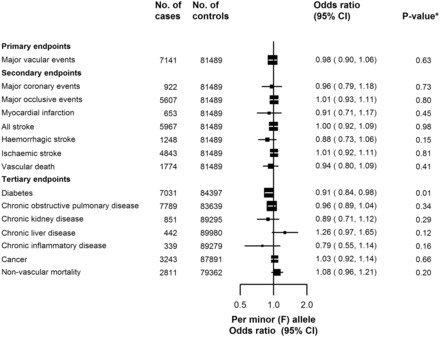
The association of *PLA2G7* V279F with vascular^19^ and non-vascular diseases. Adjusted for sex, study region, age and relatedness. Squares represent the odds ratio (OR) per Lp-PLA^2^-lowering minor (F) allele, with area inversely proportional to the variance of the log OR. Horizontal lines represent the corresponding 95% confidence intervals (CI). *P-values are not adjusted for multiple testing. Bonferroni correction based on one test (primary endpoint) or seven tests (secondary or tertiary endpoints) would result in thresholds of 0.05 (*P* = 0.05/1) or 0.007 (*P* = 0.05/7), respectively.

**Figure 2.**
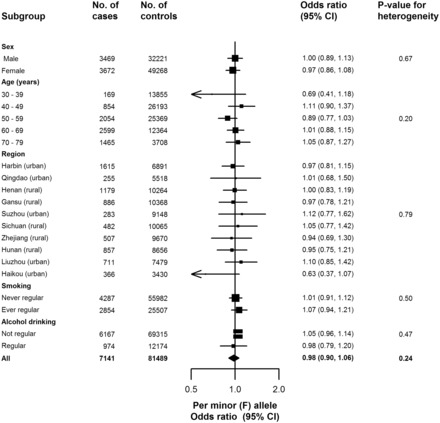
The association of *PLA2G7* V279F with major vascular events, among subgroups. Adjusted for sex (apart from sex subgroups), study region (apart from region subgroups), age (apart from age subgroups) and relatedness. Squares represent the odds ratio (OR) per Lp-PLA^2^ lowering minor (F) allele, with area inversely proportional to the variance of the log OR. Horizontal lines represent the corresponding 95% confidence intervals (CI). The diamond represents the overall OR and its 95% CI.

Figure 1 compares the risk of incident reported MVE associated with *PLA2G7* V279F among 7141 cases and 81 489 controls without vascular disease at baseline or during follow-up. As our preliminary report showed,^19^ there was no association of *PLA2G7* V279F with MVE with an OR per minor (F) allele of 0.98 (95% CI 0.90–1.06, *P* = 0.63). Although *PLA2G7* V279F showed an association with regular alcohol drinking, a possible confounder, adjusting for drinking status did not change the results (0.98, 0.91–1.07). Furthermore, there was no difference in association with MVE between subgroups defined by sex, 10-year age group, region, ever regular smoking status or current regular drinking (Figure 2).

The loss-of-function variant of *PLA2G7* V279F was not associated with components of MVE: total stroke (5967 events; 1.00, 0.92–1.09); myocardial infarction (653 events; 0.91, 0.71–1.17); and vascular death (2139 events; 0.92, 0.80–1.06). Nor was there an association with major coronary events (MCE; 922 events; 0.96, 0.79–1.18), major occlusive events (5607 events; 1.01, CI 0.93–1.11), ischaemic stroke (4843 events; 1.01, 0.92–1.11), or haemorrhagic stroke (1248 events; 0.88, 0.73–1.06). Adjustment for drinking status did not change the association of MCE with *PLA2G7* V279F (0.98, 0.80–1.21). Exploratory analyses assessed the effect of adding coronary revascularization events to the coronary endpoints, which increased the number of events but did not change the association with *PLA2G7* V279F (MCE + revascularization 1087 events; 0.95, 0.79–1.16; eTable 2, available as Supplementary data at *IJE* online). There was also no change with addition of prevalent cases of CHD, stroke or transient ischaemic attack to the incident reported vascular endpoints (MVE + previous disease 9939 events; 0.99, 0.90–1.06; eTable 2). Sensitivity analyses for MVE and MCE stratified by region followed by meta-analysis did not alter the results (data not shown).

The rs1333049 variant at the established 9p21 locus was also genotyped in study participants, and the C allele was associated with higher risk of MCE when revascularization events were included in the endpoint (1.09, 1.00–1.19, *P*-value = 0.04; eTable 3). This is consistent with previously published cohort studies (OR ranged from 1.09 to 1.13; eTable 4) and provides a positive control for genetic analyses of coronary disease in CKB.

### Association of PLA2G7 V279F with non-vascular diseases

In analyses of other chronic diseases, no associations with *PLA2G7* V279F were observed after adjustment for multiple testing (Figure 1). Among combined prevalent and new-onset cases of diabetes, whereas there was a lower risk of diabetes with *PLA2G7* V279F (*n* = 7031 events; OR = 0.91, 95% CI 0.84–0.98), there was no association after adjustment for multiple testing. There was also a lower risk of chronic inflammatory disease (0.79, 0.55–1.14), but event numbers were low (*n* = 339), resulting in a wide confidence interval. Likewise, there was no association of *PLA2G7* V279F with combined prevalent and new-onset cases of COPD, or with incident reported chronic kidney disease, chronic liver disease, cancer or non-vascular death.

Sensitivity analyses for vascular and non-vascular disease outcomes, without adjusting for first-degree relatedness, or using the subset of 82 459 unrelated individuals, demonstrated no difference between these results and estimates obtained with adjustment for relatedness in the main analyses (eFigures 2 and 3, available as Supplementary data at *IJE* online).

**Figure 3.**
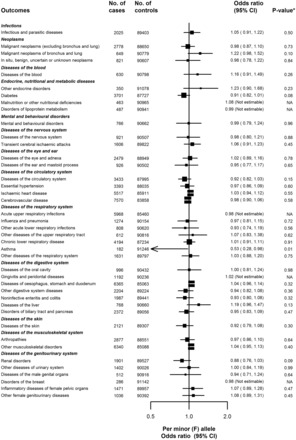
The association of *PLA2G7* V279F with ICD-10 coded disease outcomes. Conventions as in Figure 1. Missing 95% CIs indicate non-convergence of the logistic regression model due to the adjustment for relatedness, and these point estimates are not plotted. *P-values are not adjusted for multiple testing. Bonferroni correction based on 41 tests would result in a threshold of 0.001 (*P* = 0.05/41).

Among the 41 distinct disease categories in the ICD-10 coded screen, there were 196 255 coded events reported during follow-up, with 38 536 (42%) participants reporting at least one categorized event. The number of cases in each category ranged from 182 (ICD-10 J45–J46: asthma) to 7 570 (ICD-10 I60–I69: cerebrovascular disease). There was no association between *PLA2G7* V279F and any of the 41 disease categories (Figure 3). Although a reduction in risk of asthma with *PLA2G7* V279F was observed (182 events; 0.53, 0.28–0.98), there was no association after adjustment for multiple testing.

## Discussion

This is the largest single study to investigate the association of the *PLA2G7* V279F loss-of-function variant with risk of vascular diseases, and the first to investigate its effects on a wide range of disease outcomes. Among over 91 000 Chinese adults, with 11% having at least one loss-of-function variant, we found no association of *PLA2G7* V279F with major vascular or coronary events or stroke subtypes. A wide range of non-vascular outcomes were also examined, and although lower risks with *PLA2G7* V279F were observed for diabetes and asthma, there was no association after adjustment for multiple comparisons. Furthermore, there was no evidence of an association of *PLA2G7* V279F with either vascular or non-vascular death or cardiovascular risk factors. These results suggest that genetically determined lifelong lower Lp-PLA_2_ activity has no major causal effects on vascular or non-vascular diseases.

Our findings for vascular disease are consistent with null results for the association of *PLA2G7* V279F with CHD reported in meta-analyses of East Asian studies^16^^,^^17^ in which the individual studies typically involved just a few hundred cases, with diverse disease definitions, varying degrees of adjustment and little information on non-coronary diseases. Inference from the literature is often complicated by publication bias, however, since some large studies have reported only the fact of non-significance^25^ or reported results fully only in a subset with significant results,^26^ and this has distorted which results have been included in these meta-analyses. Although a Korean study reported a protective effect of V279F for myocardial infarction and angiographically defined CHD in 3700 men with adjustment for conventional risk factors (OR = 0.80, 95% CI 0.69–0.92, *P*-value = 0.002), which is not inconsistent with the 95% CIs for coronary outcomes in the present study, no effect was observed among women in the Korean study, which was attributed to possible asymptomatic CHD and misclassification among controls, and the gender-specific analyses were not pre-specified.^18^ Small case-control studies of stroke have also reported varying findings;^27–29^ however the present study, by far the largest to assess the effects of V279F on stroke, shows no evidence of benefit, particularly for ischaemic stroke. Studies of other *PLA2G7* loss-of-function variants in populations of European and African ancestry have found no evidence of effects on CHD or vascular death, although power has been limited due to low frequency of these variants.^30^^,^^31^

Results from our study are broadly in line with recent findings from randomized trials of the Lp-PLA_2_ inhibitor darapladib. The STABILITY trial (Stabilization of Atherosclerotic Plaque by Initiation of Darapladib Therapy) of 15 828 patients with stable CHD reported no effect on the primary endpoint of MVE after 3.7 years of treatment [hazard ratio (HR) 0.94, 95% CI 0.85–1.03), though a modest reduction was observed for the secondary endpoint of MCE (0.90, 0.82–1.00, *P* = 0.045).^6^ Similarly, among 13 026 ACS patients treated for 2.5 years in the randomized, double-blind, placebo-controlled trial SOLID-TIMI 52 (Stabilization of Plaque Using Darapladib-Thrombolysis in Myocardial Infarction 52), there was no effect on the primary endpoint of MCE (1.00, 0.91–1.09) or the secondary endpoint of MVE (0.99, 0.90–1.09).^7^ The trials were powered to detect a 15% reduction in relative risk, whereas lifelong exposure levels may be expected to have a greater magnitude of effect on risk than intervening to lower the exposure for just a few years in later life.^32^ Our study has ruled out any protective effect on MVE greater than 10% from lifelong exposure in this general population.

Vascular outcomes in the present study were selected to facilitate comparison with the randomized trials. However, it is notable that there was a much higher proportion of stroke events in the CKB compared with the trials, reflecting differences in cardiovascular disease rates between populations of East Asian and of European origin. Given the heterogeneity between coronary events and different stroke subtypes, use of a composite primary outcome such as MVE may hinder detection of disease-specific effects in trials as well as genetic studies, and it is important to balance the benefits of larger numbers with that of specific disease definition. Both trials reported no benefit for stroke, concordant with findings in the present study which was well powered for stroke. However, there were fewer myocardial infarction and major coronary events (*n* = 922) in the present study, and larger numbers of these events in CKB would be required to rule out relative risk reductions smaller than about 20% (odds ratio for MCE 0.96; 95% CI 0.79–1.18) to draw a definitive conclusion on these outcomes. Had the present results been available to complement existing epidemiological data when the STABILITY trial was designed,^33^ MCE might have been chosen for the primary endpoint. However, although the primary endpoint for SOLID was changed from MVE to MCE^7^ in light of the results of STABILITY, no efficacy for darapladib on either outcome was observed.

As Lp-PLA_2_ activity might play a role in multiple biological pathways, we examined the effects of *PLA2G7* V279F on a wide range of disease outcomes. Although there were nominal lower risks of diabetes and asthma, there was no evidence of association after adjusting for multiple testing. Furthermore, the direction of effect of *PLA2G7* V279F with chronic inflammatory diseases suggested a possible pro-inflammatory effect of Lp-PLA_2_ but, given the low number of events, this was not conclusive. Lp-PLA_2_ inhibitor therapies have also been investigated in phase II trials for diabetic macular oedema (NCT01506895) and Alzheimer’s disease (NCT01428453), but these outcomes were not evaluated in the present study due to insufficient numbers of reported events. The lack of increased risks of non-vascular diseases with V279F in the present study complements safety data from clinical trials of darapladib, and suggests there are no major hazards associated with lower Lp-PLA_2_ activity. Although an increase in serious renal failure events (1.5% darapladib vs 1.1% placebo; HR 1.35, 95% CI 1.03–1.78) was reported in STABILITY,^6^ we found no effect on the incidence of chronic kidney disease, though the number of acute renal failure events in the present study was too low to assess this outcome reliably. Nor was there an increased risk of cancer, in either the present study or the trials. Genetic variants influencing therapeutic targets cannot, however, be used to identify off-target drug effects.^34^

The present study has a number of strengths, including large sample size, standardized data collection, and detailed information on a wide range of disease outcomes collected through linkage to electronic health records. However, the study was still under-powered for many outcomes examined, e.g. myocardial infarction and major coronary events, which can be addressed with further genotyping of additional CKB samples and a longer follow-up period. Extrapolation from the effects of a genetic variant to the causal role of a biomarker can be hindered if the genetic variant has pleiotropic effects (i.e. other than via the biomarker) on outcomes, which are very difficult to determine. Although such effects cannot be ruled out in the present study, *PLA2G7* V279F is a functional variant close to the active domain of Lp-PLA_2_, resulting in inactive enzyme,^11^^–^^13^ and no evidence for pleiotropy was found in our analyses of baseline traits. Also, developmental compensation to a functional genetic variant could potentially alter effects on outcomes,^35^ but this is unlikely to have affected the present study as, although not directly assessed in the study participants, the loss of Lp-PLA_2_ activity in V279F carriers is well established among East Asian adults.^11^^–^^13^ Population stratification is an important consideration for Mendelian randomization studies, and although the frequency of V279F varied somewhat across the 10 CKB study regions, there was no evidence of heterogeneity of effect by region. This study, only possible in East Asians given the geographical distribution of *PLA2G7* V279F, demonstrates the value different populations can bring to Mendelian randomization investigations. The CKB is uniquely placed to investigate the role of other exposures influenced by East Asian-specific variants, such as alcohol consumption and the *ALDH2* loss-of-function variant.

### Conclusions

This study provides new evidence that lifelong lower Lp-PLA_2_ activity is unlikely to have a major causal effect on risk of vascular or non-vascular diseases in the general population, complementing findings from recent randomized trials. The use of functional genetic variants in blood-based prospective cohorts with linkage to electronic health records, such as the CKB and UK Biobank,^36^ represents a valuable approach for assessment of potential drug targets. The accumulation of further disease events over the next 5–10 years, in combination with clinical event adjudication and sub-phenotyping, will substantially increase power to detect genetic (and non-genetic) associations in the CKB. Genome-wide analysis currently ongoing in the CKB includes about 80 000 loss-of-function variants which may represent important pathways for development of novel therapies, and their effects on target outcomes can be evaluated and potential alternative indications or safety issues may also come to light. Additionally, for common, chronic diseases such as major vascular diseases which may have heterogeneous aetiology, genetically identified subtypes could help guide more efficient and targeted clinical trials. Future drug development and repositioning could benefit greatly from such information, especially if conducted in the early stages of clinical development, before undertaking large-scale clinical trials.

## Funding

This work was supported by: the Kadoorie Charitable Foundation Hong Kong; UK Wellcome Trust (grant numbers 088158/Z/09/Z, 104085/Z/14/Z); Chinese Ministry of Science and Technology (grant number 2011BAI09B01); Chinese National Natural Science Foundation (grant numbers 81390541, 81390544); GlaxoSmithKline; and Merck Sharp & Dohme Corp. The British Heart Foundation, UK Medical Research Council and Cancer Research UK provide core funding to the Clinical Trial Service Unit and Epidemiological Studies Unit at the University of Oxford. Role of the funding source: the study was part-funded by GlaxoSmithKline, who collaborated in developing the study design, analysis plan, results interpretation and reporting. All data were analysed independently at CTSU. The corresponding authors had access to all the data in the study and had final responsibility for the decision to submit for publication.

## Supplementary Material

Supplementary Data

## China Kadoorie Biobank collaborative group

International Steering Committee: Junshi Chen, Zhengming Chen (PI), Rory Collins, Liming Li (PI), Richard Peto. International Co-ordinating Centre, Oxford: Daniel Avery, Derrick Bennett, Yumei Chang, Yiping Chen, Zhengming Chen, Robert Clarke, Huaidong Du, Xuejuan Fan, Simon Gilbert, Alex Hacker, Michael Hill, Michael Holmes, Andri Iona, Rene Kerosi, Ling Kong, Om Kurmi, Garry Lancaster, Sarah Lewington, John McDonnell, Winnie Mei, Iona Millwood, Qunhua Nie, Jayakrishnan Radhakrishnan, Paul Ryder, Sam Sansome, Dan Schmidt, Paul Sherliker, Rajani Sohoni, Luanlaun Sun, Robin Walters, Jenny Wang, Lin Wang, Ling Yang, Xiaoming Yang. National Co-ordinating Centre, Beijing: Zheng Bian, Ge Chen, Lei Guo, Yu Guo, Bingyang Han, Can Hou, Peng Liu, Jun Lv, Pei Pei, Shuzhen Qu, Yunlong Tan, Canqing Yu, Huiyan Zhou. Ten Regional Co-ordinating Centres: Qingdao Qingdao CDC: Zengchang Pang, Shaojie Wang, Yun Zhang, Kui Zhang. Licang CDC: Silu Liu, Wei Hou. Heilongjiang Provincial CDC: Zhonghou Zhao, Shumei Liu, Zhigang Pang. Nangang CDC: Weijia Feng, Shuling Wu, Liqiu Yang, Huili Han, Hui He, Bo Yu. Hainan Provincial CDC: Xianhai Pan, Shanqing Wang, Hongmei Wang. Meilan CDC: Xinhua Hao, Chunxing Chen, Shuxiong Lin, Xiangyang Zheng. Jiangsu Provincial CDC: Xiaoshu Hu, Minghao Zhou, Ming Wu, Ran Tao. Suzhou CDC: Yeyuan Wang, Yihe Hu, Liangcai Ma, Renxian Zhou, Guanqun Xu, Yan Lu. Guangxi Provincial CDC: Baiqing Dong, Naying Chen, Ying Huang. Liuzhou CDC: Mingqiang Li, Jinhuai Meng, Zhigao Gan, Jiujiu Xu, Yun Liu, Jingxin Qing. Sichuan Provincial CDC: Xianping Wu, Yali Gao, Ningmei Zhang. Pengzhou CDC: Guojin Luo, Xiangsan Que, Xiaofang Chen. Gansu Provincial CDC: Pengfei Ge, Jian He, Xiaolan Ren. Maiji CDC: Hui Zhang, Enke Mao, Guanzhong Li, Zhongxiao Li, Jun He, Yulong Lei, Xiaoping Wang. Henan Provincial CDC: Guohua Liu, Baoyu Zhu, Gang Zhou, Shixian Feng. Huixian CDC: Yulian Gao, Tianyou He, Li Jiang, Jianhua Qin, Huarong Sun. Zhejiang Provincial CDC: Liqun Liu, Min Yu, Yaping Chen, Ruying Hu. Tongxiang CDC: Zhixiang Hu, Jianjin Hu, Yijian Qian, Zhiying Wu, Chunmei Wang, Lingli Chen. Hunan Provincial CDC: Wen Liu, Guangchun Li, Huilin Liu. Liuyang CDC: Xiangquan Long, Xin Xu, Youping Xiong, Zhongwen Tan, Xuqiu Xie, Yunfang Peng, Weifang Jia. GlaxoSmithKline: Lon Cardon, Stephanie Chissoe, Toby Johnson, Dawn Waterworth, Astrid Yeo. BGI: Nie Chao, Wang Jun, Qibin Li, Xiao Liu, Hongcheng Zhou.
