# Supplementary material for: A phenome-wide association study of a lipoprotein-associated phospholipase A2 loss-of-function variant in 90 000 Chinese adults
Source: Int J Epidemiol. 2016 Jun 14;45(5):1588–99. doi: 10.1093/ije/dyw087 (PMC5100610; doi:10.1093/ije/dyw087)
Supplement: Supplementary Data [file dyw087_supplementary_data.zip › ije-2015-11-1522-File006.docx]

**SUPPLEMENTARY MATERIAL**

1. Supplementary methods
2. Supplementary references
3. Supplementary tables (4)
4. Supplementary figures (3)

**SUPPLEMENTARY METHODS**

**Definition of specified endpoints and inclusion/exclusions**

*S1. Definition of ICD-10 coded study outcomes*

Major vascular events (MVE) outcome

ICD-10 I00-I99 fatal events; ICD-10 I21-I23, I60, I61, I63, I64, 196.0, I69.1, I69.3, I69.4 non-fatal events

Major coronary events (MCE) outcome

ICD-10 I20-I25 fatal events; ICD-10 I21-I23 non-fatal events

Major occlusive events outcome

ICD-10 I20-I25, I63, I69.3 fatal events; ICD-10 I21-I23, I63, I69.3 non-fatal events

Myocardial infarction (MI) outcome

ICD-10 I21-I23 fatal or non-fatal events

Stroke outcome

ICD-10 I60, I61, I63, I64, I69.0, I69.1, I69.3, I69.4 fatal or non-fatal events

Ischaemic stroke outcome

ICD-10 I63, I69.3 fatal or non-fatal events

Haemorrhagic stroke outcome

ICD-10 I61, I69.1 fatal or non-fatal events

Fatal vascular events outcome

ICD-10 I00-I99 fatal events

Diabetes outcome

ICD-10 E10-E14 fatal and non-fatal events

Chronic obstructive pulmonary disease (COPD) outcome

ICD-10 J41-J44 fatal and non-fatal events

Chronic kidney disease (CKD) broader outcome

ICD-10 E10.2+ , E11.2+, E12.2+, E13.2+, E14.2+, I12.0, I12.9, I13.0, I13.1, I13.2, I13.9, M10.3, M32.1+, N02, N03, N04, N05, N08.3*, N11, N12, N13, N15, N18, N19, N25, N26, N27.1, N27.9, N28.9, O10.2, O10.3, R94.4, T86.1, Z94.0 fatal or non-fatal events

Chronic liver disease, including cirrhosis and hepatitis (excluding alcoholic and toxic causes) outcome

ICD-10 B18, B19, B25.1+, B58.1+, B94.2, K72, K73, K74, K75, K76, K77*, Z22.5 fatal or non-fatal events

Chronic inflammatory disease outcome

ICD-10 K50, K51, L40, L88, L93, L95, M05, M06, M32, M33, M35, M45, M46

Malignant neoplasms outcome

ICD-10 C00-C97 fatal or non-fatal events

Non-vascular mortality outcome

ICD-10 A00-H95, J00-Z99 fatal events

*S2. ICD-10 coded disease categories^*^*

| ICD-10 code start | ICD-10 code end | ICD-10 code start2 | ICD-10 code end2 | ICD-10 code start3 | ICD-10 code end3 | ICD-10 code start4 | ICD-10 code end4 | Endpoint description (use or adapt ICD-10 description where possible) | Brief description |
| --- | --- | --- | --- | --- | --- | --- | --- | --- | --- |
| A00 | B99 |  |  |  |  |  |  | Certain infections and parasitic diseases | Infections |
| C00 | C33 | C37 | C99 |  |  |  |  | Malignant neoplasms (excluding bronchus and lung) | Malignant neoplasms excluding lung |
| C34 | C34 |  |  |  |  |  |  | Malignant neoplasms of bronchus and lung | Lung cancer |
| D00 | D48 |  |  |  |  |  |  | In situ, benign, uncertain or unknown behaviour neoplasms | Benign neoplasms |
| D50 | D89 |  |  |  |  |  |  | Diseases of the blood and blood-forming organs and certain disorders involving the immune mechanism | Haematological disorders |
| E00 | E07 | E15 | E35 |  |  |  |  | Disorders of thyroid gland, Other disorders of glucose regulation and pancreatic internal secretion, Disorders of other endocrine glands | Endocrine disorders excluding diabetes |
| E10 | E14 |  |  |  |  |  |  | Diabetes mellitus | Diabetes |
| E40 | E77 | E79 | E90 |  |  |  |  | Malnutrition, Other nutritional deficiencies, Obesity and other hyperalimentation, Metabolic disorders | Nutritional disorders excluding lipidaemias |
| E78 | E78 |  |  |  |  |  |  | Disorders of lipoprotein metabolism and other lipidaemias | Lipidaemias |
| F00 | F99 |  |  |  |  |  |  | Mental and behavioural disorders | Mental and behavioural disorders |
| G00 | G44 | G46 | G99 |  |  |  |  | Diseases of the nervous system (excluding TIA) | Nervous system disorders excluding TIA |
| G45 | G45 |  |  |  |  |  |  | Transient cerebral ischaemic attacks and related syndromes | Transient ischaemic attacks |
| H00 | H59 |  |  |  |  |  |  | Diseases of the eye and adnexa | Eye disorders |
| H60 | H95 |  |  |  |  |  |  | Diseases of the ear and mastoid process | Ear disorders |
| I00 | I09 | I11 | I15 | I26 | I52 | I70 | I99 | Diseases of the circulatory system (excluding hypertension, IHD, cerebrovascular disease) | Other vascular diseases |
| I10 | I10 |  |  |  |  |  |  | Essential (primary) hypertension | Hypertension |
| I20 | I25 |  |  |  |  |  |  | Ischaemic heart diseases | Ischaemic heart diseases |
| I60 | I69 |  |  |  |  |  |  | Cerebrovascular diseases | Cerebrovascular diseases |
| J00 | J06 |  |  |  |  |  |  | Acute upper respiratory infections | Acute upper respiratory infections |
| J09 | J18 |  |  |  |  |  |  | Influenza and pneumonia | Influenza/pneumonia |
| J20 | J22 |  |  |  |  |  |  | Other acute lower respiratory infections | Other acute lower respiratory infections |
| J30 | J39 |  |  |  |  |  |  | Other diseases of upper respiratory tract | Other upper respiratory tract diseases |
| J40 | J44 | J47 | J47 |  |  |  |  | Chronic lower respiratory diseases (excluding asthma) | Chronic lower respiratory diseases excluding asthma |
| J45 | J46 |  |  |  |  |  |  | Asthma, status asthmaticus | Asthma |
| J60 | J99 |  |  |  |  |  |  | Other diseases of the respiratory system | Other respiratory system diseases |
| J41 | J44 |  |  |  |  |  |  | Simple and mucopurulent chronic bronchitis, Unspecified chronic bronchitis, Emphysema, Other chronic obstructive pulmonary disease | COPD |
| K00 | K04 | K06 | K14 |  |  |  |  | Diseases of oral cavity, salivary glands and jaws (excluding gingivitis and periodontal diseases) | Oral cavity excluding periodontal |
| K05 | K05 |  |  |  |  |  |  | Gingivitis and periodontal diseases | Periodontal diseases |
| K20 | K31 |  |  |  |  |  |  | Diseases of oesophagus, stomach and duodenum | Stomach diseases |
| K35 | K46 | K55 | K67 | K90 | K93 |  |  | Diseases of appendix, Hernia, Other diseases of intestines, Diseases of peritoneum, Other diseases of the digestive system | Other digestive system diseases |
| K50 | K52 |  |  |  |  |  |  | Crohn disease [regional enteritis], Ulcerative colitis, Other noninfective gastroenteritis and colitis | Noninfective enteritis and colitis |
| K70 | K77 |  |  |  |  |  |  | Diseases of liver | Liver diseases |
| K80 | K87 |  |  |  |  |  |  | Disorders of gallbladder, biliary tract and pancreas | Gallbladder disorders |
| L00 | L99 |  |  |  |  |  |  | Diseases of the skin and subcutaneous tissue | Skin diseases |
| M00 | M25 |  |  |  |  |  |  | Arthropathies | Arthritis |
| M30 | M99 |  |  |  |  |  |  | Diseases of the musculoskeletal system and connective tissue (excluding arthropothies) | Other musculoskeletal disorders |
| N00 | N29 |  |  |  |  |  |  | Glomerular diseases, Renal tubulo-interstitial diseases, Renal failure, Urolithiasis, Other disorders of kidney and ureter | Renal disorders |
| N30 | N39 |  |  |  |  |  |  | Other diseases of urinary system | Urinary diseases |
| N40 | N51 |  |  |  |  |  |  | Diseases of male genital organs | Male genital diseases |
| N60 | N64 |  |  |  |  |  |  | Disorders of breast | Breast disorders |
| N70 | N77 |  |  |  |  |  |  | Inflammatory diseases of female pelvic organs | Female pelvic inflammatory diseases |
| N80 | N99 |  |  |  |  |  |  | Non-inflammatory disorders of female genital tract, Other disorders of the genitourinary system | Female other genitourinary diseases |

^*^Codes outside the range A00-N99 were not considered in the current study.

*S3. Inclusions and exclusions for analyses of diseases outcomes*

Vascular outcomes: Cases include participants reporting that event during follow-up, regardless of disease status at baseline. Controls include participants not reporting a physician-diagnosis of ischaemic heart disease, stroke or transient ischaemic attack at baseline, and not reporting any MVE (S1.1) during follow-up.

Diabetes: Cases include participants reporting a physician diagnosis of diabetes at baseline, participants with screen-detected diabetes based on blood glucose level at baseline, and participants reporting that event during follow-up. Controls include participants not reporting diabetes at baseline or during follow-up.

COPD: Cases include participants reporting a physician diagnosis of bronchitis or emphysema at baseline, participants with screen-detected air-flow obstruction based on spirometry measurement at baseline, and participants reporting that event during follow-up. Controls include participants not reporting COPD at baseline or during follow-up.

Chronic kidney disease, liver disease, inflammatory disease and cancer: Cases include participants reporting that event during follow-up regardless of baseline disease status. Controls include participants not reporting a physician-diagnosis of that disease at baseline.

Non-vascular mortality and ICD-10 coded disease outcomes: Cases include participants reporting that event during follow-up and controls include participants not reporting that event during follow-up, regardless of disease status at baseline

**SUPPLEMENTARY REFERENCES**

1. Lian J, Ba Y, Dai D *et al.* A replication study and a meta-analysis of the association between the CDKN2A rs1333049 polymorphism and coronary heart disease. *J Atheroscler Thromb*. 2014;**21**:1109-1120.

2. Patel RS, Asselbergs FW, Quyyumi AA *et al.* Genetic variants at chromosome 9p21 and risk of first versus subsequent coronary heart disease events: a systematic review and meta-analysis. *J Am Coll Cardiol*. 2014;**63**:2234-2245.

**eTable 1: Distribution of *PLA2G7* V279F genotype in ten study regions^a^**

| **Study region^b^** | **N** | **VV** | **VF** | **FF** | **Frequency F** | **HWE chi-square** | **HWE p-value^c^** |
| --- | --- | --- | --- | --- | --- | --- | --- |
| Harbin (Urban) | 8894 | 7837 | 1023 | 34 | 0.06 | 0.01 | 0.92 |
| Qingdao (Urban) | 5838 | 5235 | 577 | 26 | 0.05 | 5.41 | 0.02 |
| Henan (Rural) | 10 181 | 8902 | 1227 | 52 | 0.07 | 1.90 | 0.17 |
| Gansu (Rural) | 10 051 | 8952 | 1072 | 27 | 0.06 | 0.73 | 0.39 |
| Suzhou (Urban) | 7998 | 7239 | 744 | 15 | 0.05 | 0.82 | 0.37 |
| Sichuan (Rural) | 9619 | 8594 | 997 | 28 | 0.05 | 0.03 | 0.87 |
| Zhejiang (Rural) | 9123 | 8245 | 852 | 26 | 0.05 | 0.64 | 0.42 |
| Hunan (Rural) | 8883 | 8002 | 860 | 21 | 0.05 | 0.17 | 0.68 |
| Liuzhou (Urban) | 8320 | 7415 | 886 | 19 | 0.06 | 1.93 | 0.16 |
| Haikou (Urban) | 3552 | 3320 | 227 | 5 | 0.03 | 0.30 | 0.59 |
| **All regions** | **82 459** | **73 741** | **8465** | **253** | **0.05** |  |  |

^a^Allele frequencies and deviation from Hardy-Weinberg equilibrium (HWE) were assessed among the subset of 82,459 unrelated participants

^b^Regions are ordered by latitude from North to South

**^c^**P-values presented are not adjusted for multiple testing. Bonferroni correction based on 10 tests would result in a threshold of 0.005 for significance at P<0.05

**eTable 2: Association of *PLA2G7* V279F with selected vascular diseases with additional revascularisation events or prior disease cases**

| **Outcome^a^** | **No. cases** | **Odds ratio (95% CI) per minor (F) allele** | **P-trend** |
| --- | --- | --- | --- |
| Major vascular events (MVE) | 7140 | 0.98 ( 0.90, 1.06) | 0.6286 |
| MVE + revascularization | 7278 | 0.98 ( 0.90, 1.06) | 0.6168 |
| MVE + prior coronary heart disease, stroke and transient ischaemic attack | 9939 | 0.99 ( 0.93, 1.07) | 0.8767 |
|  |  |  |  |
| Major coronary events (MCE) | 922 | 0.96 ( 0.79, 1.18) | 0.7283 |
| MCE + revascularization | 1087 | 0.95 ( 0.79, 1.16) | 0.6216 |
| MCE + prior coronary heart disease | 3460 | 1.01 ( 0.90, 1.13) | 0.8911 |
|  |  |  |  |
| Stroke | 5967 | 1.00 ( 0.92, 1.09) | 0.9764 |
| Stroke + prior stroke and transient ischaemic attack | 6898 | 1.00 ( 0.92, 1.09) | 0.9709 |
|  |  |  |  |
| Major occlusive events | 5607 | 1.01 ( 0.93, 1.11) | 0.8011 |
| Major occlusive events + revascularization | 5749 | 1.01 ( 0.92, 1.10) | 0.8339 |
|  |  |  |  |
| Myocardial infarction | 653 | 0.91 ( 0.71, 1.17) | 0.4514 |
| Myocardial infarction + revascularization | 823 | 0.90 ( 0.72, 1.13) | 0.3447 |
|  |  |  |  |

^a^All analyses are adjusted for age, sex, region and relatedness

**eTable 3: Association of rs1333049 at the 9p21 locus with coronary disease without and with revascularisation events**

| **Outcome^a^** | **No. cases** | **Odds ratio (95% CI) per C allele** | **P-trend** |
| --- | --- | --- | --- |
| Major coronary events | 921 | 1.07 ( 0.98, 1.17) | 0.1505 |
| Major coronary events + revascularization | 1,085 | 1.09 ( 1.00, 1.19) | 0.0426 |
| Myocardial infarction | 652 | 1.03 ( 0.92, 1.15) | 0.6338 |
| Myocardial infarction + revascularization | 821 | 1.07 ( 0.97, 1.18) | 0.2003 |

^a^All analyses are adjusted for age, sex, region and relatedness

**eTable 4: Comparison of 9p21 association between CKB and published studies in European and East Asian populations**

| **Study design and population** | **Outcome** | **No. cases** | **Odds ratio (95% CI) per copy of risk allele** |
| --- | --- | --- | --- |
| *Case-control studies* |  |  |  |
| European meta-analysis^1^ | Coronary heart disease | 24 126 | 1.30 (1.16, 1.47) |
| East Asian meta-analysis^1^ | Coronary heart disease | 17 044 | 1.27 (1.22, 1.33) |
| *Prospective cohort studies* |  |  |  |
| European meta-analysis^2^ | Fatal/nonfatal myocardial infarction | 2293 | 1.09 (1.03, 1.16) |
|  | Myocardial infarction, death, unstable angina, revascularization, hospitalisation | 5880 | 1.13 (1.08, 1.17) |
| East Asian (China Kadoorie Biobank unpublished) | Fatal coronary heart disease, fatal/nonfatal myocardial infarction | 921 | 1.07 (0.98, 1.18) |
|  | Fatal coronary heart disease, fatal/nonfatal myocardial infarction, revascularization | 1085 | 1.09 (1.00, 1.19) |

**SUPPLEMENTARY FIGURES**

**eFigure 1.** **Study participant flowchart**

**eFigure 2.** **The association of *PLA2G7* V279F with vascular and non-vascular diseases without adjustment for relatedness**

Sensitivity analyses adjusted for sex, study region and age. Squares represent the odds ratio (OR) per Lp-PLA_2_-lowering minor (F) allele, with area inversely proportional to the variance of the log OR. Horizontal lines represent the corresponding 95% confidence intervals (CI). P-values are not adjusted for multiple testing. Bonferroni correction based on 1 test (primary endpoint), or 7 tests (secondary or tertiary endpoints) would result in thresholds of 0.05 (P=0.05/1) or 0.007 (P=0.05/7).

**eFigure 3. The association of *PLA2G7* V279F with vascular and non-vascular diseases among unrelated participants**

Sensitivity analyses conducted among 82 459 unrelated participants. Conventions as in eFigure 2.


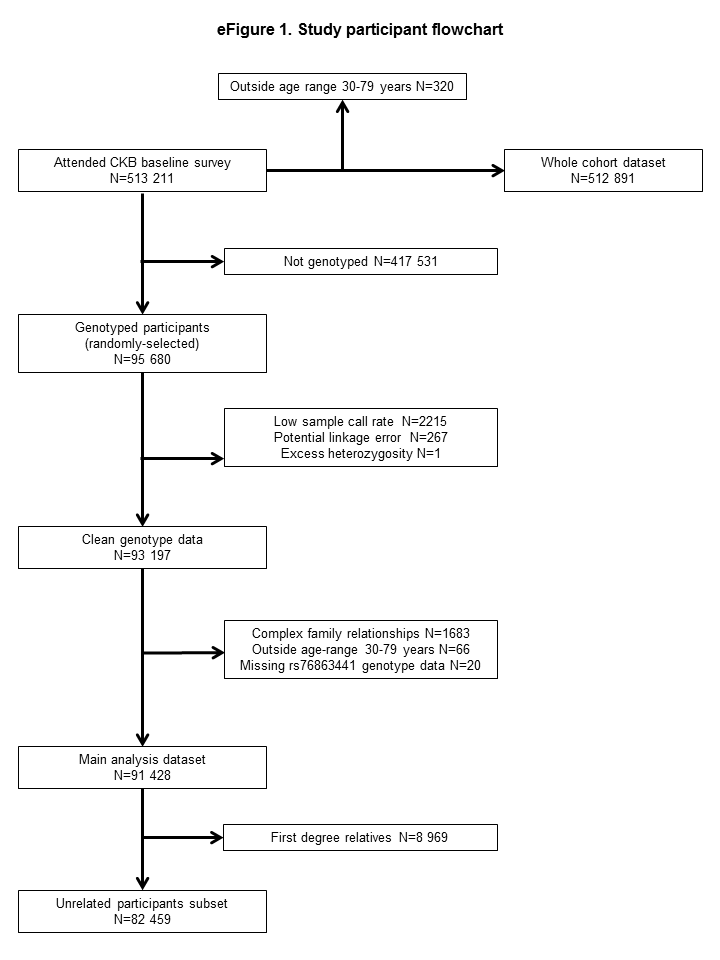


**
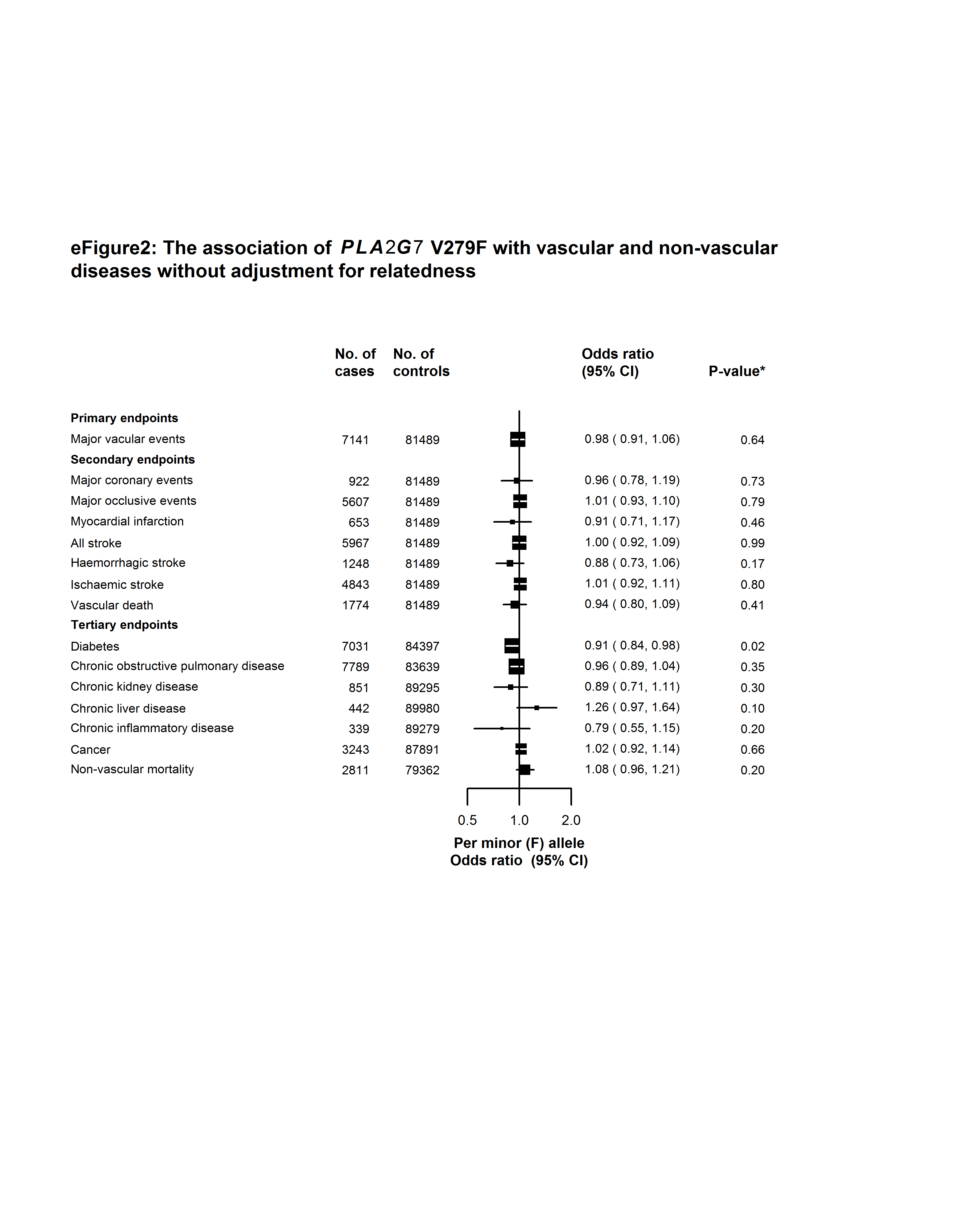
**

**
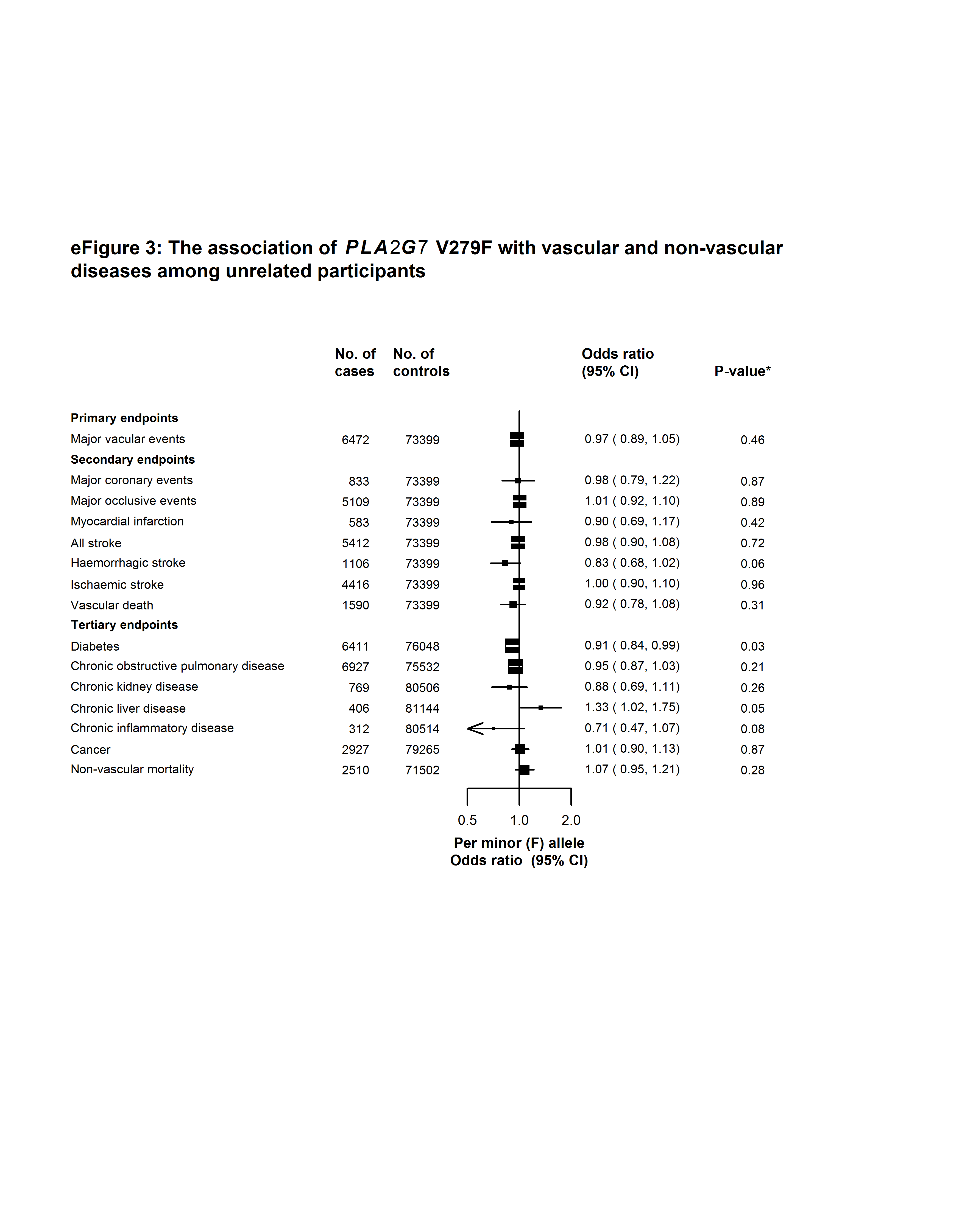
**
